# Impact of preoperative patient education on the prevention of postoperative complications after major visceral surgery: the cluster randomized controlled PEDUCAT trial

**DOI:** 10.1186/s13063-018-2676-6

**Published:** 2018-05-24

**Authors:** Ulla Klaiber, Lisa M. Stephan-Paulsen, Thomas Bruckner, Gisela Müller, Silke Auer, Ingrid Farrenkopf, Christine Fink, Colette Dörr-Harim, Markus K. Diener, Markus W. Büchler, Phillip Knebel

**Affiliations:** 1 Department of General, Visceral, and Transplantation Surgery, University of Heidelberg, Heidelberg, Germany; 2 Institute of Medical Biometry and Informatics, University of Heidelberg, Heidelberg, Germany

**Keywords:** Patient education, Preoperative education, Postoperative complication, Prevention, Visceral surgery, Cluster randomization

## Abstract

**Background:**

The prevention of postoperative complications is of prime importance after complex elective abdominal operations. Preoperative patient education may prevent postoperative complications and improve patients’ wellbeing, but evidence for its efficacy is poor. The aims of the PEDUCAT trial were (a) to assess the impact of preoperative patient education on postoperative complications and patient-reported outcomes in patients scheduled for elective complex visceral surgery and (b) to evaluate the feasibility of cluster randomization in this setting.

**Methods:**

Adult patients (age ≥ 18 years) scheduled for elective major visceral surgery were randomly assigned in clusters to attend a preoperative education seminar or to the control group receiving the department’s standard care. Outcome measures were the postoperative complications pneumonia, deep vein thrombosis (DVT), pulmonary embolism, burst abdomen, and in-hospital fall, together with patient-reported outcomes (postoperative pain, anxiety and depression, patient satisfaction, quality of life), length of hospital stay (LOS), and postoperative mortality within 30 days after the index operation. Statistical analysis was primarily by intention to treat.

**Results:**

In total 244 patients (60 clusters) were finally included (intervention group 138 patients; control group 106 patients). Allocation of hospital wards instead of individual patients facilitated study conduct and reduced confusion about group assignment. In the intervention and control groups respectively, pneumonia occurred in 7.4% versus 8.3% (*p* = 0.807), pulmonary embolism in 1.6% versus 1.0% (*p* = 0.707), burst abdomen in 4.2% versus 1.0% (*p* = 0.165), and in-hospital falls in 0.0% versus 4.2% of patients (*p* = 0.024). DVT did not occur in any of the patients. Mortality rates (1.4% versus 1.9%, *p* = 0.790) and LOS (14.2 (+/− 12.0) days versus 16.1 (+/− 15.0) days, *p* = 0.285) were also similar in the intervention and control groups.

**Conclusions:**

Cluster randomization was feasible in the setting of preoperative patient education and reduced the risk of contamination effects. The results of this trial indicate good postoperative outcomes in patients undergoing major visceral surgery without superiority of preoperative patient education compared to standard patient care at a high-volume center. However, preoperative patient education is a helpful instrument not only for teaching patients but also for training the nursing staff.

**Trial registration:**

German Clinical Trials Registry, DRKS00004226. Registered on 23 October 2012. Registered 8 days after the first enrollment.

**Electronic supplementary material:**

The online version of this article (10.1186/s13063-018-2676-6) contains supplementary material, which is available to authorized users.

## Background

Major visceral resection is the treatment of choice for various benign and malignant diseases of the visceral organs and often represents the only chance for cure in cancer patients. Recent advances in surgical techniques and perioperative management have enabled expert surgeons to perform highly demanding and extended operations with acceptable mortality rates in specialized institutions. However, overall postoperative morbidity remains at 24–44%, depending on the definitions used, the type of operations performed, and the patients’ characteristics [1–3]. Postoperative complications considerably impair patients’ postoperative outcome, lengthening intensive care unit and total hospital stay and increasing mortality [3]. In view of the large numbers of operations carried out worldwide and the cost increases caused, postoperative complications burden not only the individual patient but also the healthcare system [4]. Thus, the prevention of postoperative complications is of prime importance.

Not all risk factors for the development of postoperative complications can be controlled during the postoperative period. For instance, numerous patient-related factors, e.g., gender, age, and body mass index, and procedure-related factors, e.g., tumor localization and surgical site, cannot be changed in the acute postoperative situation. In this context, it is of particular importance to be aware of preventable risk factors and to fully exploit the potential to control them. Deep vein thrombosis (DVT), pulmonary embolism, pneumonia, burst abdomen, and in-hospital falls are frequent and important postoperative complications that can be limited to some extent by preventive measures to reduce associated risk factors such as postoperative pain, coughing, and atelectasis [5–7]. Patients’ knowledge of and ability to perform preventive exercises in the postoperative period is considered a logical prerequisite for active prevention of the aforementioned complications. However, surgeons’ lack of time for extensive patient visits and the concept of fast-track surgery make it difficult to give sufficient information to patients. In these circumstances, a preoperative patient education seminar carried out by nursing staff seems promising. Studies assessing the impact of preoperative patient education, covering teaching of skills and psychological support, on patients’ recovery, postoperative pain, and psychological distress go back more than 20 years [8]. However, the evidence on preoperative patient education is still inconsistent and the benefit-expenditure ratio remains a matter of debate [9, 10]. In particular, evidence for preoperative patient education in patients undergoing major visceral surgery is sparse. Cluster randomization offers the possibility to allocate hospital wards instead of individual patients to the study groups, which may guarantee adherence to group assignment and facilitate the conduct of a randomized controlled trial addressing preoperative patient education.

Therefore, the aims of the PEDUCAT trial were (a) to investigate the impact of preoperative patient education on the postoperative complications pneumonia, DVT, pulmonary embolism, burst abdomen, in-hospital fall, and on mortality, postoperative pain, perioperative anxiety and depression, quality of life (QoL), and length of hospital stay (LOS) in patients undergoing major visceral surgery, and (b) to evaluate the feasibility of a cluster randomized trial in this setting. The objectives of the PEDUCAT trial pertained primarily to the individual patient level.

## Methods

**Fig. 1:**
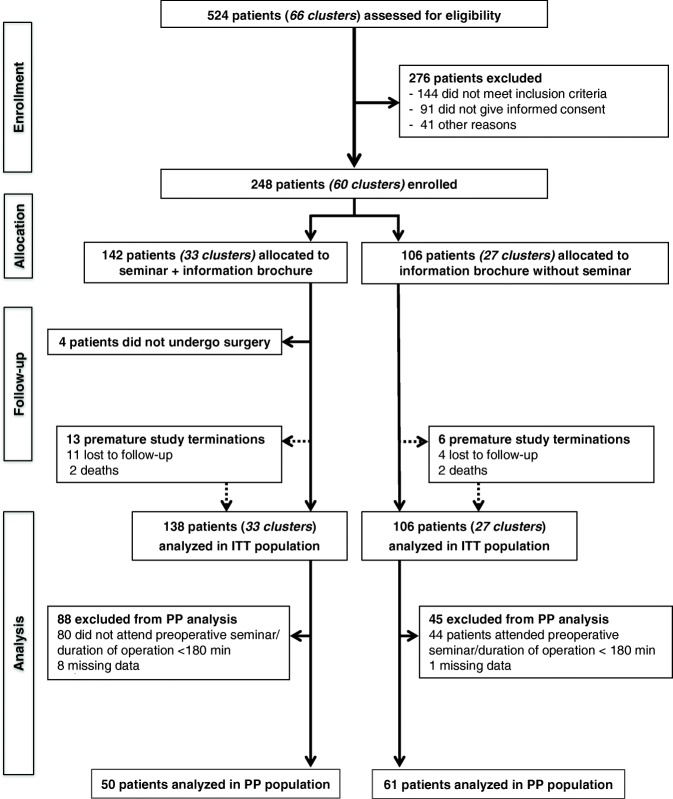
Flow diagram. PP, per protocol; ITT, intention to treat

This trial was conducted by the Clinical Study Center (KSC) together with the nursing staff of the Department of General, Visceral and Transplantation Surgery, University of Heidelberg, with the support of the Institute of Medical Biometry and Informatics (IMBI), University of Heidelberg. The study protocol was approved by the ethics committee of the University of Heidelberg on 2 October 2012 (S-376/2012) and then published [9]. The study was registered with the German Clinical Trials Registry (DRKS00004226) on 23 October 2012. The study adhered to the recommendations of the updated and extended Consolidated Standards of Reporting Trials (CONSORT) Statement for the reporting of cluster randomized trials [11]. The CONSORT checklist is provided as Additional file 1, and a flow diagram is presented in Fig. 1.

### Participants

PEDUCAT was a cluster randomized, controlled, single-center pilot trial with two parallel study groups. Eligible participants were patients ≥ 18 years of age scheduled for elective major visceral surgery, defined as a surgical procedure with a planned operating time ≥ 180 min. Only patients electively admitted to one of two predefined wards of the Department of General, Visceral and Transplantation Surgery, University of Heidelberg during the period Monday to Wednesday were eligible. The exclusion criteria were impaired mental capacity, language barrier, physical constraints, infections requiring isolation, and previous participation in the preoperative patient education program at Heidelberg University Hospital. Individual patients were included in the trial after giving written informed consent. Consent was sought after randomization.

### Procedures

The trial intervention was a preoperative patient education seminar given by qualified nursing staff on the day before surgery. During this standardized 1-h event the patients (who were part of a cluster) learned about measures to prevent postoperative complications, with the focus on pneumonia, thrombosis, pulmonary embolism, burst abdomen, and in-hospital fall. Furthermore, they were instructed about the principles of acute pain therapy and various coping strategies, e.g., autogenic training and progressive muscle relaxation. Patients were introduced to breathing exercises, careful postoperative out-of-bed mobilization, and practical exercises to prevent thrombosis and burst abdomen. The risks for and preventive measures against in-hospital fall were also explained. In addition to the seminar, patients received the center’s standard 48-page information brochure, which included the same topics. In the control group, patients (who were part of another cluster) received only the information brochure and the standard communications with the surgeon and ward nurses.

For the assessment of patient data and outcomes, five study visits were performed as described in detail in the published study protocol [9]. The study visits were documented on paper-based case report forms. Data were captured prospectively in the database. Serious adverse events (SAE) were documented on specific forms and reported to the principal investigator within 7 days.

### Cluster randomization

Patients were randomly assigned in clusters to the intervention or control group. A cluster was defined as all patients electively admitted to one of two predefined wards of the Department of General, Visceral and Transplantation Surgery, University of Heidelberg during the period Monday to Wednesday. A computer-generated list prepared by the IMBI, University of Heidelberg, was used for randomization. The randomization list was in the charge of an independent member of staff of the KSC who disclosed the randomization of the two wards every 2 weeks in order to prevent possible contamination effects. The investigators were then informed about the randomization result. Participants were enrolled in clusters and informed about their group allocation by the investigators. Permuted-block randomization with an allocation ratio of 1:1 and a block size of 4 was used. The intra-cluster correlation coefficient and design effect were calculated to determine the effect of clustering on the outcomes analyzed.

### Outcomes

Outcome measures comprised the postoperative complications of pneumonia, DVT, pulmonary embolism, burst abdomen, and in-hospital fall within 30 days after the index operation and a composite endpoint summarizing these complications. Detailed definitions of all endpoints are provided in the study protocol [9]. Other complications were recorded via the SAE documentation when severity fulfilled criteria for grade IV or V complications according to Clavien-Dindo [12]. Additionally, LOS (time from day of operation until hospital discharge) and postoperative 30-day mortality were assessed. The questionnaires, the Brief Pain Inventory (BPI) [13], Hospital Anxiety and Depression Scale (HADS) comprising the subscales for anxiety (HADS-A) and depression (HADS-D) [14], and the 12-item Short Form Survey (SF-12) [15], were completed by the patients themselves for the assessment of postoperative pain, perioperative anxiety and depression, and QoL, respectively. Additionally, patients completed an unvalidated questionnaire comprising four items on a 5-point scale (ranging from 1 to 5) assessing patient satisfaction. Three additional items addressed the preoperative seminar. Moreover, the feasibility of cluster randomization was evaluated.

### Statistical analysis

As usual in exploratory study designs, no formal sample size calculation was performed. To test the feasibility of patient recruitment and cluster randomization it was planned to randomize 204 patients (34 clusters). This sample size would have been able to show a treatment effect of 18% or more for the composite endpoint, at a two-sided α-level of 5% and with power of 90%. The primary analysis was based on the intention-to-treat (ITT) population to represent clinical practice. Additionally, a per-protocol (PP) analysis was performed on those patients in the intervention group who attended the preoperative seminar and subsequently underwent complex surgery (i.e., duration of operation ≥ 180 min) and those patients in the control group who did not participate in the seminar but underwent complex surgery (i.e., duration of operation ≥ 180 min). Depending on the scale level of the variables, means and standard deviations or absolute and relative frequencies were calculated. To compare the treatment groups, corresponding statistical tests were performed and descriptive *p* values were calculated. A *p* value less than 0.05 was considered to show a statistically significant difference. To account for the cluster randomization multilevel regression was performed with patients at level 1 and education cluster at level 2 (GLIMMIX procedure). Calculations were performed using SAS software (version 9.3; SAS Institute, Inc., Cary, NC, USA). In the case of missing data, patients were excluded from statistical analysis of the outcome measure concerned. Due to the nature of the trial all reported *p* values have to be treated as descriptive statistics without confirmatory value.

## Results

### Recruitment period and intention-to-treat population

Between 15 October 2012 and 5 February 2014, a total of 248 patients were randomly assigned in 60 clusters to the intervention or the control group (Fig. 1). Four patients in the intervention group had to be excluded from analysis as the operation was canceled after randomization. The ITT analysis population thus consisted of 244 patients (intervention group *n* = 138, control group *n* = 106) and 60 clusters. As the allocation of the participants to one of the two study groups shows, cluster randomization ensured a balanced distribution of patients to the two groups in terms of baseline characteristics. Sex, age, body mass index, American Society of Anesthesiologists (ASA) score, target organ in the operation, comorbidities, and length of the operation were distributed equally in the two groups (Table 1). Overall, in 102 patients the duration of surgery was less than 180 min (intervention group *n* = 60, control group *n* = 42). The main reasons for the operating time being shorter than planned were irresectability of a malignant tumor and the performance of a palliative procedure, e.g., gastric or biliary bypass, instead of the planned resection. Allocation of hospital wards instead of individual patients facilitated the study conduct and reduced confusion about group assignment. Nevertheless, 34 out of 138 patients (24.6%) in the intervention group did not attend the preoperative seminar, whereas 2 out of 106 patients (1.9%) in the control group participated in the seminar. Main reasons for patients in the intervention group not attending the preoperative seminar were competing appointments for preoperative preparations.

**Table 1:** Baseline characteristics of the intention-to-treat analysis population

	Patient education + information brochure (*n* = 138)	Information brochure only (*n* = 106)
Sex		
Male	74 (53.6%)	56 (52.8%)
Female	64 (46.4%)	50 (47.2%)
Age (years)	57.0 (14.0)	56.8 (13.4)
Body mass index (kg/m^2^)	25.1 (4.2)	25.7 (5.2)
ASA classification		
I (normal healthy patient)	7 (5.1%)	4 (3.8%)
II (mild systemic disease)	72 (52.2%)	60 (56.6%)
III (severe systemic disease)	58 (42.0%)	41 (38.7%)
IV (constant threat to life)	1 (0.7%)	1 (0.9%)
Target organ for operation		
Esophagus	3 (2.2%)	4 (3.8%)
Stomach	9 (6.5%)	5 (4.7%)
Small intestine	5 (3.6%)	6 (5.7%)
Colon	19 (13.8%)	14 (13.2%)
Rectum	9 (6.5%)	8 (7.5%)
Pancreas	59 (42.8%)	39 (36.8%)
Liver	12 (8.7%)	13 (12.3%)
Kidney	9 (6.5%)	6 (5.7%)
Other	13 (9.4%)	11 (10.4%)
Comorbidities		
Chronic cardiovascular disease	41 (29.7%)	36 (34.0%)
Chronic pulmonary disease	15 (10.9%)	12 (11.3%)
History of deep vein thrombosis	10 (7.2%)	13 (12.3%)
History of pulmonary embolism	1 (0.7%)	5 (4.7%)
Diabetes mellitus	21 (15.2%)	9 (8.5%)
Other significant disease	66 (47.8%)	48 (45.3%)
Previous laparotomy (incision > 10 cm)	59 (42.8%)	39 (36.8%)
Duration of operation (min)	211.9 (116.9)	216.3 (110.7)

Data are means (with standard deviations) or numbers of patients (with percentages)

*ASA* American Society of Anesthesiologists

The results of this cluster randomized trial show an intra-cluster correlation coefficient of 0.01, resulting in an design effect of 1.04 when applying to a mean cluster size of five patients per cluster.

### Postoperative complications

In the intervention group 13 out of 120 patients (10.8%) and in the control group 12 out of 96 patients (12.5%) had at least one of the five predefined postoperative complications (pneumonia, DVT, pulmonary embolism, burst abdomen, or in-hospital fall) within 30 days after the index operation. There was no statistically significant difference between the two study groups (*p* = 0.704). Furthermore, no significant difference was found on direct comparison of the single items of the composite endpoint (Table 2), with the exception of in-hospital fall which occurred only in the control group (in 4 out of 96 patients, 4.2%; *p* = 0.024).

**Table 2:** Postoperative morbidity and mortality within 30 days and length of hospital stay

	Patient education + information brochure (*n* = 138)	Information brochure only (*n* = 106)	*P* value
Postoperative morbidity			
Pneumonia			0.807*
*N*	121	96	
*n*	9 (7.4%)	8 (8.3%)	
Deep vein thrombosis			Not applicable
*N*	120	96	
*n*	0 (0.0%)	0 (0.0%)	
Pulmonary embolism			0.707*
*N*	122	96	
*n*	2 (1.6%)	1 (1.0%)	
Burst abdomen			0.165*
*N*	120	96	
*n*	5 (4.2%)	1 (1.0%)	
In-hospital fall			0.024*
*N*	120	96	
*n*	0 (0.0%)	4 (4.2%)	
Composite endpoint^a^			0.704*
*N*	120	96	
*n*	13 (10.8%)	12 (12.5%)	
Mortality			0.790*
*N*	138	106	
*n*	2 (1.4%)	2 (1.9%)	
Length of hospital stay (days)			0.285^†^
*N*	138	106	
*n*	14.2 (12.0)	16.1 (15.0)	

Significant results (*p* value < 0.05) are in bold

Data are means (with standard deviations) or numbers of patients (with percentages)

^a^Patients with at least one complication

^*^χ^2^ test

^†^*t* test

*N* Number of patients available for the outcome analyzed

*n* Number of events

With two deaths in each group, the mortality rates were similar (*p* = 0.790). There was no significant difference between the two study groups in LOS. The mean duration of stay was 14.2 (± 12.0) days in the intervention group and 16.1 (± 15.0) days in the control group (*p* = 0.285) (Table 2).

### Patient-reported outcomes

**Fig. 2:**
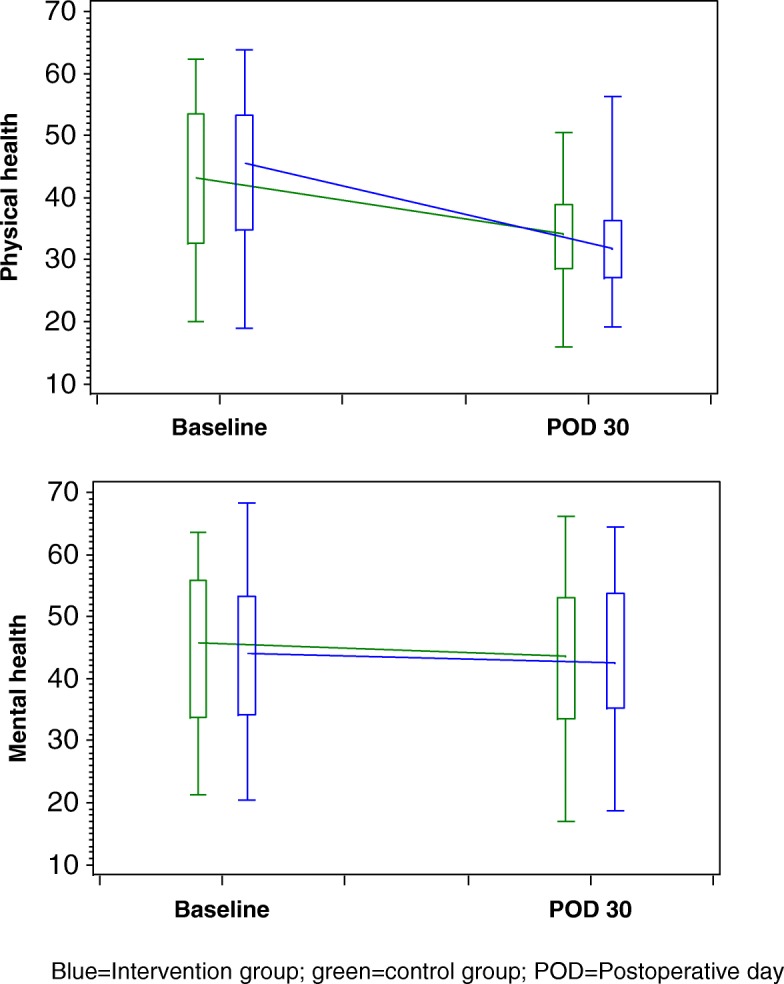
Postoperative course of quality of life. Blue, intervention group; green, control group; POD, postoperative day

**Fig. 3:**
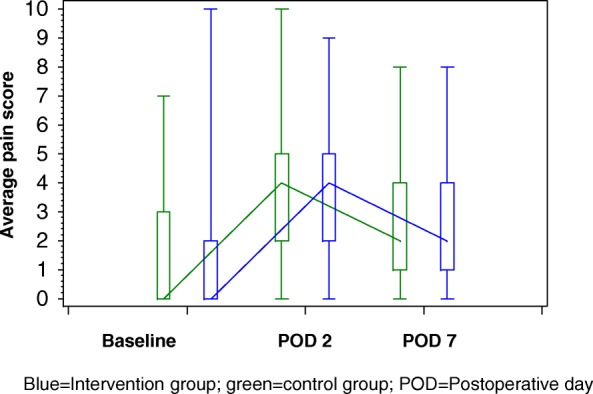
Postoperative course of pain. Blue, intervention group; green, control group; POD, postoperative day

**Fig. 4:**
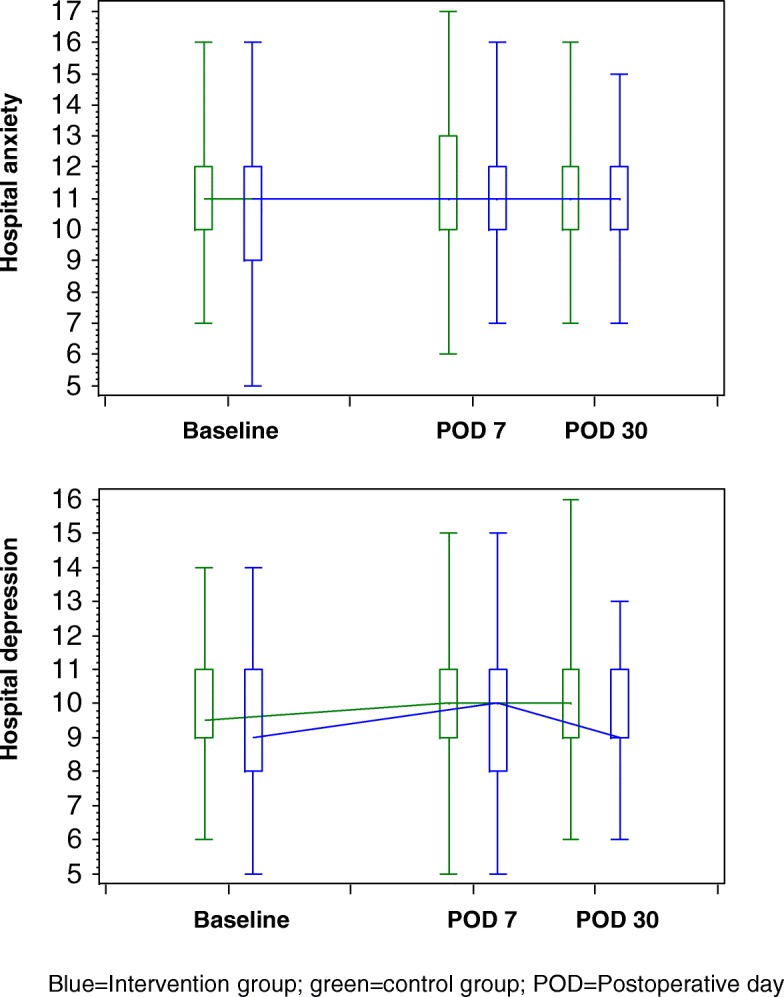
Postoperative course of anxiety and depression. Blue, intervention group; green, control group; POD, postoperative day

The results of the self-completed questionnaires for the assessment of patient-reported outcomes and patient satisfaction are presented in Tables 3, 4, 5 and 6. The physical and mental health QoL scores in the two groups were similar at baseline and on postoperative day (POD) 30 (Table 3 and Fig. 2). QoL was worse in both study groups 30 days after the index operation than it was before surgery, with a greater decline in physical health scores than in mental health scores (Fig. 2). Pain scores were comparable between the two groups before and after surgery (i.e., on POD 2 and POD 7) with the following exception: on POD 7, the intensity of pain at the time of completing the questionnaire was significantly higher in the intervention group than in the control group (*p* = 0.023) (Table 4). In both study groups, average pain scores reached their maximum on POD 2 and declined afterwards (Fig. 3). There was no statistically significant difference between the two groups in anxiety and depression at baseline and on POD 7 (Table 5 and Fig. 4). However, depression values were significantly higher in the control group than in the intervention group on POD 30 (*p* = 0.049), while anxiety values were similar on POD 30 (*p* = 0.479). As shown in Fig. 4, median values of perioperative anxiety stayed constant at a score of 11 during the perioperative course in both groups. In contrast, perioperative depression reached a maximum on POD 7 in both groups and declined thereafter in the intervention group, but not in the control group (Fig. 4).

**Table 3:** Quality of life

	Patient education + information brochure (*n* = 138)	Information brochure only (*n* = 106)	*P* value
At baseline			
Short Form (SF)-12 physical health score			0.298^†^
*N*	116	89	
Mean (±SD)	44.0 (±10.9)	42.4 (±11.3)	
SF-12 mental health score			0.514^†^
*N*	116	89	
Mean (±SD)	43.4 (±11.6)	44.5 (±12.0)	
At 30 days after operation			
SF-12 physical health score			0.179^†^
*N*	82	68	
Mean (±SD)	32.2 (±7.3)	33.9 (±7.5)	
SF-12 mental health score			0.848^†^
*N*	82	68	
Mean (±SD)	43.4 (±11.7)	43.8 (±12.4)	

^†^*t* test

**Table 4:** Postoperative pain

	Patient education + information brochure (*n* = 138)	Information brochure only (*n* = 106)	*P* value
At baseline			
Pain at its worst in the past 24 h.			0.859*
*N*	136	105	
Mean (±SD)	1.9 (±2.7)	1.8 (±2.5)	
Pain on average in the past 24 h.			0.958*
*N*	136	104	
Mean (±SD)	1.4 (±2.0)	1.4 (±2.0)	
Pain right now			0.820*
*N*	136	105	
Mean (±SD)	1.1 (±1.8)	1.1 (±1.8)	
In the past 24 h, pain has interfered with ...			
... mood			0.404*
*N*	136	105	
Mean (±SD)	1.7 (±2.5)	1.6 (±2.5)	
... walking ability			0.505*
*N*	135	105	
Mean (±SD)	1.0 (±1.9)	0.8 (±1.6)	
... relation with other people			0.749*
*N*	136	105	
Mean (±SD)	0.9 (±1.8)	1.0 (±2.0)	
... sleep			0.776*
*N*	135	105	
Mean (±SD)	2.1 (±2.9)	2.1 (±3.0)	
... concentration			0.929*
*N*	136	104	
Mean (±SD)	1.5 (±2.2)	1.6 (±2.3)	
At 2 days after operation			
Pain at its worst in the past 24 h			0.515*
*N*	131	95	
Mean (±SD)	5.7 (±2.8)	5.9 (±2.8)	
Pain on average in the past 24 h			0.559*
*N*	131	95	
Mean (±SD)	3.6 (±2.2)	3.8 (±2.1)	
Pain right now.			0.759*
N	131	95	
Mean (±SD)	2.5 (±2.3)	2.5 (±2.5)	
In the past 24 h, pain has interfered with ...			
... mood			0.463*
*N*	131	93	
Mean (±SD)	3.7 (±3.4)	4.1 (±3.4)	
... walking ability			0.293*
*N*	131	93	
Mean (±SD)	5.0 (±3.5)	5.4 (±3.4)	
... relation with other people			0.939*
*N*	127	92	
Mean (±SD)	2.4 (±3.0)	2.4 (±3.0)	
... sleep			0.423*
*N*	131	94	
Mean (±SD)	3.4 (±3.3)	3.9 (±3.5)	
... concentration			0.752*
*N*	131	93	
Mean (±SD)	3.4 (±3.2)	3.5 (±3.4)	
At 7 days after operation			
Pain at its worst in the past 24 h			0.835*
*N*	121	88	
Mean (±SD)	4.1 (±2.9)	4.1 (±2.7)	
Pain on average in the past 24 h			0.487*
*N*	121	88	
Mean (±SD)	2.8 (±2.0)	2.6 (±1.9)	
Pain right now			0.023*
*N*	121	87	
Mean (±SD)	2.3 (±1.9)	1.8 (±2.0)	
In the past 24 h, pain has interfered with ...			
... mood			0.705*
*N*	120	86	
Mean (±SD)	3.3 (±2.9)	3.2 (±3.1)	
...walking ability			0.433*
*N*	121	87	
Mean (±SD)	3.0 (±2.5)	3.4 (±3.0)	
... relation with other people			0.202*
*N*	119	87	
Mean (±SD)	2.1 (±2.4)	1.7 (±2.4)	
... sleep			0.747*
*N*	119	88	
Mean (±SD)	3.7 (±2.8)	3.6 (±3.0)	
... concentration			0.894*
*N*	120	88	
Mean (±SD)	2.8 (±2.7)	2.9 (±3.1)	

Significant results (*p* value < 0.05) are in bold

^*^U test

**Table 5:** Perioperative anxiety and depression

	Patient education + information brochure (*n* = 138)	Information brochure only (*n* = 106)	*P* value
At baseline			
Hospital anxiety scale			0.527^†^
*N*	135	103	
Mean (±SD)	10.7 (±1.7)	10.8 (±1.9)	
Hospital depression scale			0.297^†^
*N*	135	104	
Mean (±SD)	9.5 (±1.9)	9.7 (±1.6)	
At 7 days after operation			
Hospital anxiety scale			0.389^†^
*N*	118	88	
Mean (±SD)	11.1 (±1.7)	11.3 (±2.0)	
Hospital depression scale			0.202†
*N*	119	88	
Mean (±SD)	9.7 (±1.8)	10.0 (±2.0)	
At 30 days after operation			
Hospital anxiety scale			0.479^†^
*N*	100	78	
Mean (±SD)	10.9 (±1.5)	11.0 (±1.6)	
Hospital depression scale			**0.049** ^**†**^
*N*	100	78	
Mean (±SD)	9.6 (±1.5)	10.0 (±1.5)	

Significant results (*p* value < 0.05) are in bold

^†^*t* test

**Table 6:** Patient satisfaction

	Patient education + information brochure (*n* = 138)	Information brochure only (*n* = 106)	*P* value
“I have been informed about the correct behavior after the operation sufficiently”			0.473*
*N*	117	88	
Mean (±SD)	1.8 (±1.0)	1.8 (±1.0)	
“I feel insecure and I am afraid of doing something wrong”			0.911*
*N*	118	87	
Mean (±SD)	3.1 (±1.4)	3.1 (±1.4)	
“Before the operation, I had enough possibilities to address my worries”			0.683*
*N*	118	87	
Mean (±SD)	1.9 (±1.1)	1.9 (±1.1)	
“I felt sufficiently prepared for the operation”			0.567*
*N*	115	88	
Mean (±SD)	1.7 (±1.0)	1.6 (±1.0)	
“The preoperative education has influenced postoperative recovery in a positive way”			Not applicable
*N*	86	Not available	
Mean (±SD)	1.8 (±0.8)		
“The preoperative education has encouraged me to take part in postoperative recovery actively”			Not applicable
*N*	86	Not available	
Mean (±SD)	1.7 (±0.9)		
“The preoperative patient education was important for me”			Not applicable
*N*	86	Not available	
Mean (±SD)	1.7 (±0.9)		

Unvalidated questionnaire: 1 = completely agree, 5 = not correct at all

^*^U test

Both in the intervention and in the control group, patients were highly satisfied with the information they received about correct behavior after the operation, with the opportunity to address their worries preoperatively, and with being prepared for the operation, and there was no statistically significant difference between the two groups in these items. Additionally, patients’ feelings of insecurity and fear of doing something wrong were moderate and comparable in both groups. The vast majority of patients in the intervention group judged the preoperative patient education as an important factor affecting postoperative recovery in a positive way. Furthermore, most patients stated that the preoperative education seminar had encouraged them to take an active part in their postoperative recovery and judged the seminar as important for them (Table 6).

### Serious adverse events (SAE)

There was no significant difference between the two groups in the occurrence, severity, causality, and outcome of SAE (Table 7).

**Table 7:** Serious adverse events

	Patient education + information brochure (*n* = 138)	Information brochure only (*n* = 106)	*P* value
Occurrence of SAE	13 (9.4%)	10 (9.4%)	0.997*
Severity			0.772*
Grade IV*	11 (84.6%)	8 (80.0%)	
Grade V*	2 (15.4%)	2 (20.0%)	
Causality			Not applicable
Unrelated	13 (100%)	10 (100%)	
Possibly related	0 (0%)	0 (0%)	
Definitely related	0 (0%)	0 (0%)	
Outcome			0.361*
Recovered completely	3 (23.1%)	3 (30.0%)	
Recovered with sequelae	4 (30.8%)	2 (20.0%)	
Death	2 (15.4%)	2 (20.0%)	
Unknown	1 (7.7%)	3 (30.0%)	
Ongoing	3 (23.1%)	0 (0%)	

Data are numbers of patients (with percentages)

*SAE* serious adverse events

^*^χ^2^ test

### Per-protocol analysis

After exclusion of a total of 133 patients from analysis, the PP analysis population consisted of 111 patients, with 50 patients in the intervention group and 61 patients in the control group (Fig. 1). PP analysis did not substantially alter the results, except for the following endpoints: in contrast to the ITT analysis showing significantly more in-hospital falls in the control group, the occurrence of in-hospital falls was similar in the two study groups on PP analysis (intervention group 0% versus control group 3.4%, *p* = 0.208). Moreover, again in contrast to the ITT analysis, neither the intensity of pain on POD 7 (*p* = 0.168) nor depression values on POD 30 (*p* = 0.102) were significantly different between the two groups. In comparison to the ITT analysis, which showed no significant difference between the intervention and control groups, PP analysis revealed significantly lower maximum pain values in the intervention group on POD 2 (*p* = 0.007) and significantly lower degrees of interference of pain with walking ability and sleep in the intervention group on POD 2 (*p* = 0.006 and 0.024, respectively).

## Discussion

This is the first cluster randomized controlled trial evaluating the impact of preoperative patient education on postoperative outcomes in patients undergoing major visceral surgery. The results show that except for in-hospital falls, the incidence of the postoperative complications analyzed was similar in the two study groups, with low frequencies in each. Our data on burst abdomen are in line with recent reports of burst abdomen in 1–5% of patients undergoing complex abdominal surgery [2, 16]. Postoperative pneumonia is a common complication following abdominal surgical operations, with reported incidences ranging from 0.5 to 28% [17]. The highest frequencies are observed in patients undergoing esophagectomy, due to the elevated risk of aspiration after this procedure. In comparison, the incidence of pneumonia in the PEDUCAT trial, below 9%, is rather low, even though 2.9% of the patients included in this trial underwent esophagectomy. According to a current retrospective cohort analysis evaluating a total of 33,325 patients undergoing abdominal surgery, DVT and pulmonary embolism occur in less than 1% of patients [18]. The authors attribute the low frequencies to improvements in prophylaxis adherence. In the PEDUCAT trial, DVT was not observed in any patient. This may be primarily explained by the relatively small sample size. In contrast, at up to 1.6%, the frequency of pulmonary embolism was higher than reported in the literature. However, unlike the PEDUCAT trial, Wang et al. [18] excluded patients with underlying malignancy - a known risk factor for pulmonary embolism [19]. Furthermore, they examined only operations of low to moderate complexity (colectomy, enterectomy, hysterectomy, and abdominal wall hernia repair), whereas pancreatic resections represent the most commonly performed operations in the PEDUCAT trial. The results on hospital falls in this study are comparable with reports of frequencies below 2% within 30 days of the operation [20].

In the PEDUCAT trial, cluster randomization was used to allocate hospital wards rather than individual patients to the intervention and control groups. As a result, confusion about group allocation could be avoided and adherence to study group was ensured in the majority of patients. The main reason for the few patients in the intervention group not attending the preoperative seminar was competing appointments for preoperative preparations. Coincidence of appointments might be reduced in future trials by better scheduling. With an intra-cluster correlation coefficient of 0.01 the effect of clustering on the outcomes measured was rather low in the PEDUCAT trial. With regard to future RCT planning the sample size is to be increased appropriately in prospective trials on the same issue to account for the clustering (design effect = 1.04). Based on our experience, cluster randomization is recommended whenever group adherence might be jeopardized by individual randomized trial designs. Moreover, cluster randomization is an adequate tool to prevent contamination effects (e.g., when patient X of the treatment group shares the same room with patient Y of the control group, the treatment effect is expected to disappear).

Results on the postoperative complications of pneumonia, DVT, pulmonary embolism, and burst abdomen were similar in both the intervention and control groups in this study. This is important, as the incidence of these postoperative complications can be influenced to some extent by preventive measures. On ITT analysis the occurrence of in-hospital falls was significantly higher in the control group than in the intervention group. However, this difference was not significant in the PP analysis. On the other hand, the planned sample size of 204 patients may have been too small to show a statistically significant difference between the two groups. Only a difference of 18% or more in the composite endpoint would have been statistically significant, while the real difference was below 2%.

Contrary to other studies investigating preoperative patient education [21, 22], patients in the PEDUCAT trial were highly and comparably satisfied in both study groups, with no significant differences in their perception of pain, QoL, or anxiety and depression. This may be attributable to the randomized design of this trial, minimizing relevant sources of bias, e.g., selection bias, compared with several previous studies featuring retrospective and non-randomized designs [8]. Additionally, results from studies investigating the effects of preoperative patient education in an ambulatory setting cannot be generalized [21, 22]. The effect of the intervention may become smaller in major surgery, where the quality of surgical and nursing procedures plays a greater part. In this context, and in line with the literature [23], the good patient-reported outcomes may be attributable to the low morbidity and mortality rates in this study and thus be independent of the study group.

In interpreting of the results of this study, it has to be taken into account that a patient education seminar teaches not only the patients but also the nursing staff. Importantly, the patient education seminars were performed by the same nurses who took care of the patients on the wards, which may have had an additional positive impact on the quality of patient care in daily practice independent of the study groups. This effect may even have been the reason for non-significance of the trial results. Therefore, the conduct of a patient education seminar may be an important means of guaranteeing high-quality patient care, even though superiority could not be measured. On the other hand, the duration and timing of the seminar may be inadequate to achieve a benefit which means that one hour on the day before surgery may be too late and too short. However, as patients are not admitted to hospital until the day before surgery, it seems to be the most practicable day. To solve the problem, the information brochure could be sent to the patients several days before the operation with the aim of informing the patients in advance and to prepare them for the seminar. Therewith, the theoretical knowledge might be applied during the seminar and the patients might build on their existing knowledge. Depending on the patients’ preexisting knowledge and demands, the duration of the seminar could be extended to guarantee that every patient is well-prepared for the operation.

Due to the exploratory and open-label design of the study, several limitations have to be taken into account. First, the results are prone to performance and detection bias, so the results of self-completed questionnaires assessing pain, QoL, and perioperative anxiety and depression must be interpreted with caution. In contrast, the postoperative outcomes of pneumonia, DVT, pulmonary embolism, burst abdomen, and in-hospital fall are objective - so-called “hard” - outcome parameters, which are less prone to bias [24]. Another shortcoming of this trial is that because of delayed operations and/or prolonged hospital stays in some cases, a contamination effect in a few patients cannot be excluded. Consequently, randomization intervals should be extended in future trials. Furthermore, owing to the monocentric trial design, generalizability of data is limited.

## Conclusions

The results of this study indicate that certain postoperative complications such as pneumonia, DVT, pulmonary embolism, burst abdomen, and in-hospital fall following major abdominal surgery may be prevented by professional patient care. A preoperative patient education seminar may be beneficial in training both the patients and the nursing staff and should thus be offered to patients scheduled for complex abdominal surgery. However, to prove equivalence or non-inferiority of such a seminar a further cluster randomized controlled trial with a confirmatory and multicenter study design would be necessary.

## Additional file

Additional file 1: CONSORT checklist. (DOCX 36 kb)

## Abbreviations

ASA: American Society of Anesthesiologists
BPI: Brief Pain Inventory
CONSORT: Consolidated Standards of Reporting Trials
DRKS: German Clinical Trials Registry
DVT: Deep vein thrombosis
HADS: Hospital Anxiety and Depression Scale
IMBI: Institute of Medical Biometry and Informatics
ITT: Intention to treat
KSC: Clinical Study Center
LOS: Length of hospital stay
POD: Postoperative day
PP: Per protocol
QoL: Quality of life
SAE: Serious adverse event
SF-12: 12-item Short Form Survey
